# Effect of Harvesting Stage on Sweet Sorghum (*Sorghum bicolor* L.) Genotypes in Western Kenya

**DOI:** 10.1155/2017/8249532

**Published:** 2017-02-01

**Authors:** Moses Owuor Oyier, James O. Owuoche, Maurice E. Oyoo, Erick Cheruiyot, Betty Mulianga, Justice Rono

**Affiliations:** ^1^Department of Crops, Horticulture and Soils, Egerton University, P.O. Box 536, Egerton, Kenya; ^2^Kenya Agriculture and Livestock Research Organization, Sugar Research Institute, P.O. Box 44, Kisumu, Kenya; ^3^Department of Biochemistry and Molecular Biology, Egerton University, P.O. Box 536, Egerton, Kenya

## Abstract

Harvesting stage of sweet sorghum (*Sorghum bicolor* L. Moench) cane is an important aspect in the content of sugar for production of industrial alcohol. Four sweet sorghum genotypes were evaluated for harvesting stage in a randomized complete block design. In order to determine sorghum harvest growth stage for bioethanol production, sorghum canes were harvested at intervals of seven days after anthesis. The genotypes were evaluated at different stages of development for maximum production of bioethanol from flowering to physiological maturity. The canes were crushed and juice fermented to produce ethanol. Measurements of chlorophyll were taken at various stages as well as panicles from the harvested canes. Dried kernels at 14% moisture content were also weighed at various stages. Chlorophyll, grain weight, absolute ethanol volume, juice volume, cane yield, and brix showed significant (*p* = 0.05) differences for genotypes as well as the stages of harvesting. Results from this study showed that harvesting sweet sorghum at stages IV and V (104 to 117 days after planting) would be appropriate for production of kernels and ethanol. EUSS10 has the highest ethanol potential (1062.78 l ha^−1^) due to excellent juice volume (22976.9 l ha^−1^) and EUSS11 (985.26 l ha^−1^) due to its high brix (16.21).

## 1. Introduction

Sweet sorghum (*Sorghum bicolor* L.) is an indigenous C4 crop to Africa and is a propoor multipurpose crop providing food, feed, fiber, and fuel across a range of agroecosystems [1]. In Kenya, sweet sorghum has the potential to improve the food security situation by provision of food and feed as well as supply of cane in sugarcane industries for ethanol production [2]. Sorghum is a multipurpose crop well adapted to environmental conditions ranging from tropical to temperate conditions within 40°N and 40°S of the equator [3]. The potential of sorghum is enormous because the panicle can be harvested to obtain kernel which can be used for food while the stalk can be harvested for both fodder and fuel production [4].

It is necessary to determine stage of harvesting sweet sorghum for kernel, fodder, and biofuel production [5]. Stage of harvesting sweet sorghum cane for ethanol production is important to farmers as well as food, fodder, and biofuel industries [5]. Studies have been done on sweet sorghum sugar traits but those concerned with the harvesting stage of sweet sorghum relevant to its food, fodder, and fuel utilities are minimal [6–10]. Tsuchihashi and Goto [11] worked on harvesting time of sweet sorghum and found that it was optimum at the hard dough stage but did not consider the effect of various stages of harvesting on the properties of sugar and quality of kernels. It is worth mentioning that biofuel industry is constantly competing with food supply globally because many biofuel plant sources are the same sources that supply food to ever increasing human population [12]. In USA, tonnes of maize* (Zea mays)* and sorghum are used annually for production of biofuels [13]. This has elicited arguments concerning the competition between using food products as biofuels and using them to enhance food security in some developing countries [14]. Most populations in such countries still suffer from hunger and malnutrition due to lack of food thus necessitating a careful balance between energy and food security interventions [15].

The need to grow obligate cash crops such as sugarcane competes unfavorably with food production due to stiff competition on the arable land hence complicating the food security situation especially in developing countries [16]. R. Paarlberg and R. L. Paarlberg [17] argued that if a balance between the growing of cash crops and food crops is not made then countries remain at the brink of famine [17]. Cognizant of this fact, growing of multipurpose crops such as sweet sorghum and assessment of the effect of harvesting stage on the quality of the grain and fuel which can be obtained is not only important but also essential for the resource poor farmers who can maximize their gain in sweet sorghum farming. Thus the assessment of the harvesting stage of sweet sorghum can help farmers to know when to harvest their sorghum crop depending on their economic important parts and a balance for the maximum benefit [18]. In the USA, 6000 L ha^−1^ of ethanol has been produced from sweet sorghum cane but this production is low compared to the quantity obtained from sugarcane [19]. However, even though the ethanol yield per unit weight of feedstock is lower for sweet sorghum cane compared to sugarcane, the low production costs and water requirement for this crop compensates for the difference and returns a competitive cost advantage in the production of ethanol [20].

The physiological size and activities of sink organ influence the competitive ability to import photoassimilates [21]. Sugar accumulation in the stems of sweet sorghum is a function of metabolism and transport processes in the plant [22]. For this reason, to increase stem sucrose content of sweet sorghum it is necessary to select for large stem sizes. A sweet sorghum cultivar, Keller, had been developed and has high performance in a wide range of environmental conditions in the USA [23]. Currently, there are efforts globally to promote the production of biofuel from sweet sorghum cane [24]. In Australia, sweet sorghum is grown in South Eastern Queensland and canes are supplied to the biofuel industry to produce industrial alcohol while, in the USA, sweet sorghum is used for ethanol and fodder production [25, 26]. Studies based on the harvesting stage of sweet sorghum were only concerned with sugar accumulation in the cane but not maximization of the grain as well [10–12]. Consequently, information on the appropriate stage of harvesting for sweet sorghum is limited. The knowledge on useful qualities of sweet sorghum genotypes and their stage of harvesting is necessary for farmers who may want to target economic and by products from the crop [5].

Chlorophyll level indicates the photosynthetic activity of the plant and can be used to predict maturity and harvesting stage of sweet sorghum cane. High chlorophyll content in the leaves is related to high photosynthetic activity and vice versa and detection of content of chlorophyll through remote sensing can be used to predict appropriate harvesting stage for production of alcohol. However, Zarco-Tejada et al. [26] observed that low concentration of chlorophyll content in the leaves is an indicator of nitrogen deficiency and may indicate false maturity in the crop [26]. To avoid false detection, Haboudane et al. [27] suggested that the precision of agricultural practices can be increased by remote sensing. Care should be taken on the utilization of this method. It is necessary to set a baseline and investigate soil nutrient conditions before using this approach [28]. Objectives of this study were to determine the effect of harvesting stage of sweet sorghum cane from four genotypes on the fuel alcohol and to investigate the content of chlorophyll on the leaves of sweet sorghum at different harvesting stages.

## 2. Materials and Method

### 2.1. Genotypes

Three sweet sorghum lines (EUSS10, EUSS11, and EUSS17) and one cultivar, SS04, were used in this study. SS04 is a cultivated sweet sorghum variety while the other three are under development by Egerton University.

### 2.2. Experimental Site and Environmental Conditions

The experiment was conducted at Kibos, Sugar Research Institute experimental fields (0°04′06′′S, 34°49′03′′E), with an altitude of 1173 m above sea level, about 8 Km East of Kisumu City, in the western part of Kenya. This area experiences mean precipitation of 1323 mm per annum with the onset of long rains in March while short rains commence in August with a gradual reduction towards September and about 374.4 mm in December. In general, the average maximum temperature of this location is 30°C with a minimum of 15.5°C. The soils in the experimental site are predominantly heavy black cotton soil.

### 2.3. Experimental Procedure

Four sweet sorghum genotypes were planted at Sugar Research Institute (SRI) experimental plots. The field that was under maize cultivation in the previous season was disc ploughed and harrowed twice to achieve a fine tilth suitable for planting sorghum. The experiment was conducted in Randomized Completely Block Design (RCBD) with four replications. Within the replicates, sorghum genotypes were planted at seeding rate of 10 Kg ha^−1^ in an experimental unit measuring 3 m × 5 m with interrow spacing of 60 cm and intraspacing of 15 cm. At planting time, each plot received an equivalent rate of 30 Kg ha^−1^, 10 Kg ha^−1^ of K, and 40 Kg N ha^−1^. At growth stage, plants were thinned to one plant per hole; six weeks after planting, additional 40 Kg N ha^−1^ was supplied to each plot. Infestation by shoot fly* (Atherigona soccata)* on young seedlings was minimized by spraying a systematic insecticide Bulldock (*beta-cyfluthrin* 25 gl^−1^) at 25 g ha^−1^ at intervals of 14 days for one month. Within the experimental plots, weed growth was restricted by mechanical weeding. Weeding and intercultivation operations were done twice between 5 leaves and panicle emerges from the boot. Between booting stage and the end of anthesis, a second dose of Bulldock was applied at 25 g ha^−1^ to control sorghum midge* (Contarinia sorghicola)*. After heading, the panicles were covered using paper bags to protect from bird damage. Harvesting of sweet sorghum canes was done from the onset of flowering (50% flowering) to maturity (Table 1).

### 2.4. Data Collection

From a sample of three plants, the mean heights of the plants were determined at two levels when 50% of the plants had flowered in order to determine the overall height and the height at the flag leaf. In addition, mean girth was determined from a sample of 3 plants using Vanier calipers at the fifth internode. From each entry, a sample of 5 plants per plot from the middle row were harvested by cutting the plant at the base and stripped, at an interval of 7 days for 5 weeks.

Stalk juice was extracted in three-roller crusher (Fuan Liyuan, China, type YC 80B-4) once and strained through a 1 mm sieve into a glass juice container and filtered to remove large particles. The wet bagasse weight was determined immediately using Ashton Meyer's digital balance. Thereafter, brix in the juice samples was estimated using refractometer.

Single drop of juice from each sample was dropped on the hand-held refractometer to estimate the brix. Juice extraction (%) was computed by dividing weight of fresh juice by weight of fresh stalks and multiplying by 100. Juice yield was computed by multiplying average juice weight from 5 plants by plants per hectare. Determination of cane yield, juice yield, brix, and ethanol was done at an interval of 7 days for five weeks. A further investigation was done on the brix with the view of understanding its rate of accumulation. This was done by fitting curves and equation of the curves, finding their first derivatives of the equations and plotting their functions to obtain parabolic curves (Table 2). Fermentation of the juice was done by adding approximately 1.5 g yeast* (Saccharomyces cerevisiae)* to 100 ml of juice sample and incubating at 35°C for 4 days. The fermented samples were then distilled by heating in a conical flask connected to a Liebig condenser and the ethanol content in the distillates was determined by measuring the refractive indices on a hand-held refractometer (model: standard line Alla made in France). Determinations were based on a standard curve drawn by measuring the refractive indices of absolute ethanol solutions (0, 5, 10, 15, 20, 25, and 30%) in distilled water.

Chlorophyll level in the leaves was determined from the 50% flowering stage and through all stages of harvesting, using a SPAD 502 chlorophyll meter. The chlorophyll level was determined from the leaf on the 5th node from the ground and at the mid section of the leaf for consistency. The 5th leaf also happens to have the largest surface area and remains attached to the plant till maturity. It was obtained from all plants marked for harvesting and the average chlorophyll level was determined. Five plants were randomly selected per plot and harvested by cutting at the base which is the middle of the first internode. For each of the harvested plants, the panicles were removed and air dried (25°C) for 21 days. Thereafter, cane yield, excluding the leaves, was determined from the stalks. The average yield in mass was determined for every stage of harvesting for each genotype after drying them to approximately 14% moisture content.

## 3. Results and Discussion

### 3.1. Environmental Conditions

**Figure 1:**
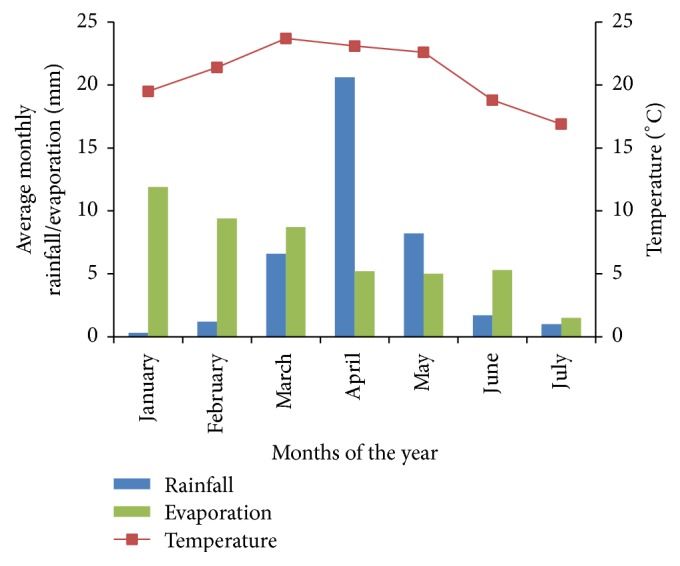
Weather data for the period January to July 2016 from SRI meteorological station about 200 m from the experimental field.

The growing period commenced from 14th April 2016 to July 2016. At the time of planting on 14th April, The seedbed was saturated with moisture after receiving 471.4 mm of rainfall. April was the month with the highest rainfall in the growing season with moderate rainfall experienced in May (254.9 mm) and June (52 mm) (Figure 1). Relatively low temperatures were experienced with April mean at 16.9°C, May at 17°C, and June at 16°C. Harvesting was done in July and during this period low temperatures (15°C) and rainfall (4 mm) were experienced (Figure 1).

### 3.2. Analysis of Variance of Sweet Sorghum Traits

There were significant (*p* ≤ 0.05) differences due to genotype for overall height, height at the flag leaf, diameter of the girth, juice volume, brix, ethanol, chlorophyll, and grain weight (Table 3). Significant (*p* ≤ 0.05) effects due to stage and genotype × stage interaction were detected for cane yield, girth, juice volume, brix, ethanol, chlorophyll, and grain weight. Although significant effects due to genotype and stage of harvesting were observed, no significant effects due to genotype × stage of harvesting interactions were observed for cane yield. There was significant variation between genotypes for days to 50% flowering. EUSS17 took the least (57 days) to flower followed by SS04 (69 days) and EUSS11 (73 days) but it took 82 days for EUSS10 to attain anthesis stage. Line EUSS10 attained an overall height of 182 cm, followed by EUSS17 (179 cm) and EUSS11 (175 cm). Cultivar SS04 attained a height of 188 cm. However, harvesting stage did not influence height for the sorghum lines tested. The average deviation between the overall height and the height at the flag leaf was 27.5 cm, with the highest deviation of 37 cm detected on line SS04.

Among the four genotypes, there was significant difference (*p* ≤ 0.05) in the total cane yield per hectare between EUSS10 and EUSS11 but there was no significant difference among EUSS11 and EUSS17 and EUSS17 and SS04 (Table 3). It is worth noting that sorghum line EUSS10 depicted the highest cane yield of 57 tonnes ha^−1^ at harvesting stage III. Both EUSS17 and SS04 produced similar yields for most of the stages of harvesting (Figure 3) but EUSS17 exhibited the lowest cane yield at all the stages of harvesting. Although harvesting stages I, II, and III were not significantly different for cane yield, there were significant differences (*p* = 0.05) between harvesting stages IV and VI (Table 4).

**Figure 2:**
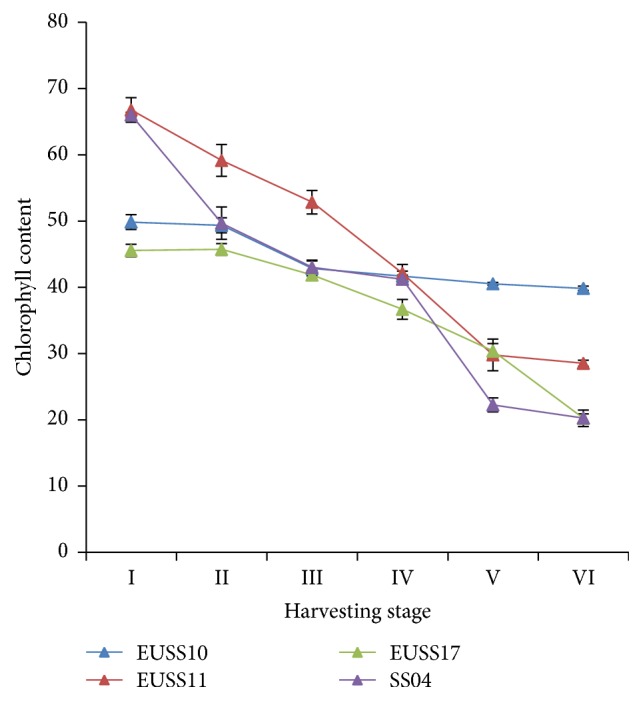
Effect of harvesting stage on chlorophyll content of four sweet sorghum genotypes evaluated in part of western Kenya.

**Figure 3:**
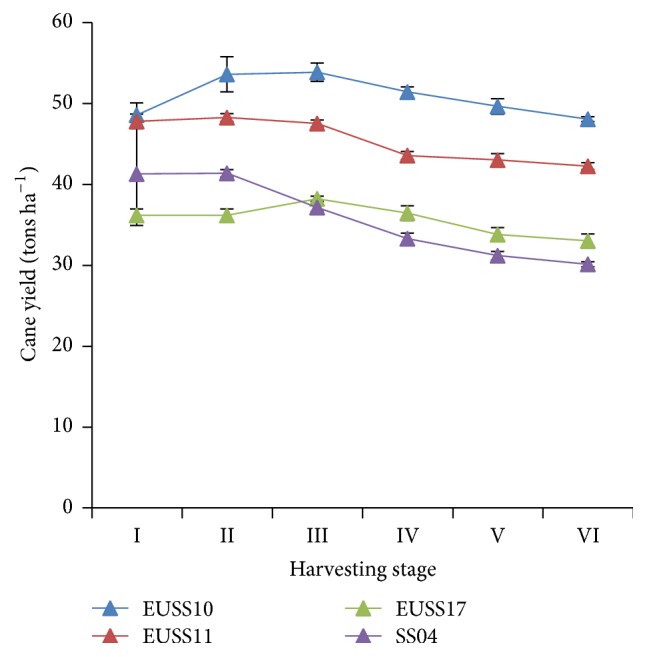
Effect of harvesting stage on cane yield of four sweet sorghum genotypes evaluated in western part of Kenya.

In this study, concentration of chlorophyll decreased from the highest in the first stage of harvesting to the lowest in the last stage of harvesting (Figure 2). The highest level of chlorophyll was observed at the first stage which was 57, 69, 73, and 82 days after sowing for EUSS17, SS04, EUSS11, and EUSS10, respectively. In general, there was a steady reduction of chlorophyll content as sorghum matures. Among the four sorghum genotypes, EUSS10 had the highest chlorophyll content. From analysis of linear regression on the level of chlorophyll in the leaves, it was evident that SS04 had the highest rate of decrease of chlorophyll at −8.93 (Table 5). This was followed by EUSS11 and EUSS17 at −8.2871 and −5.823, respectively (Table 5). The lowest rate of reduction of chlorophyll concentration was observed on sorghum line EUSS10 at −2.2166 as well as the lowest* y*-intercept (Table 5).

**Figure 4:**
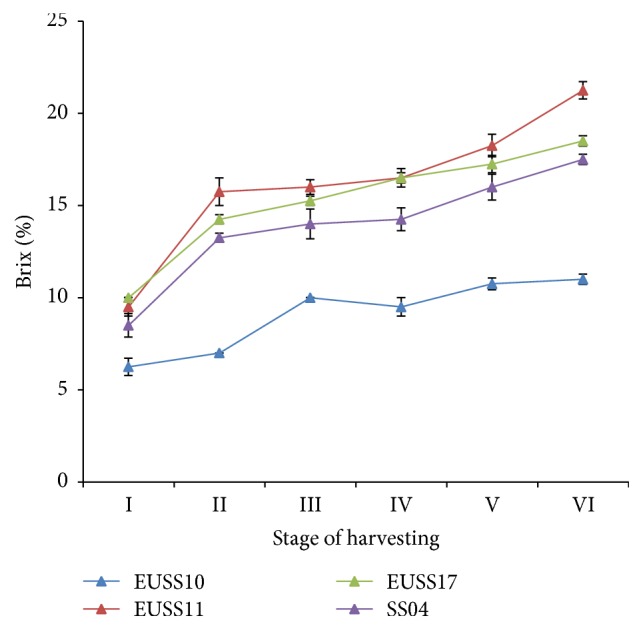
Effect of harvesting stage on brix (%) of four sweet sorghum genotypes in western part of Kenya.

There was significant difference (*p* ≤ 0.05) in the brix for all genotypes except for EUSS17 and SS04 (Table 4) and for all the stages of harvesting except for stages III and IV (Table 4). The genotypes, the stages of harvesting and their interaction showed significant variation in brix accumulation (Table 3). Brix is a good indicator of the maturity of the crop for harvesting for ethanol production. EUSS11 and EUSS17 had the highest brix at all the harvesting stages (Figure 4). EUSS10 had low brix and the increase was slower and almost stagnant at stages V and VI of harvesting. Furthermore, the highest rate of brix accumulation was seen for genotype EUSS11 which was at the rate of 1.9 after every 7 days. This was followed by SS04, EUSS17, and EUSS10 at 1.53, 1.5, and 0.98, respectively (Table 5).

**Figure 5:**
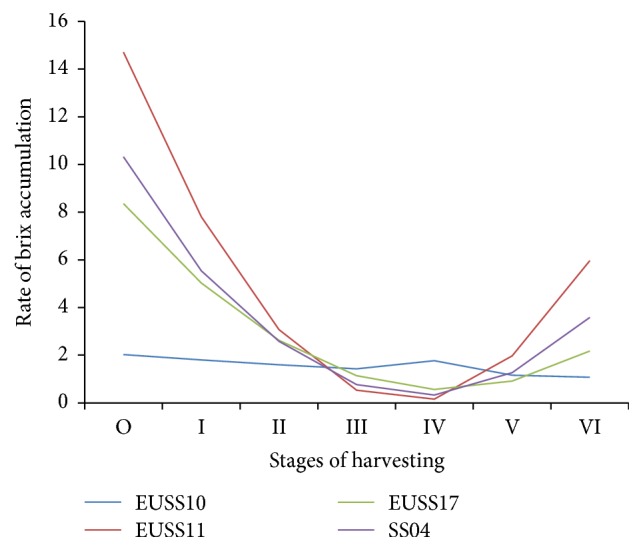
Rate of accumulation of brix against the stages of harvesting among the four genotypes, a polynomial of degree 3.

There was rapid decrease of brix in sorghum line for EUSS11, EUSS17, and SS04 up to stage III; then there was a steady increase up to stage VI. EUSS10 showed very gradual decrease for all the stages of harvesting (Figure 5). For EUSS11, EUSS17, and SS04, the parabolas indicated that the rates of brix accumulation were dropping from stage I to stage III and then started increasing at increasing rate again from stage IV to stage VI.

**Figure 6:**
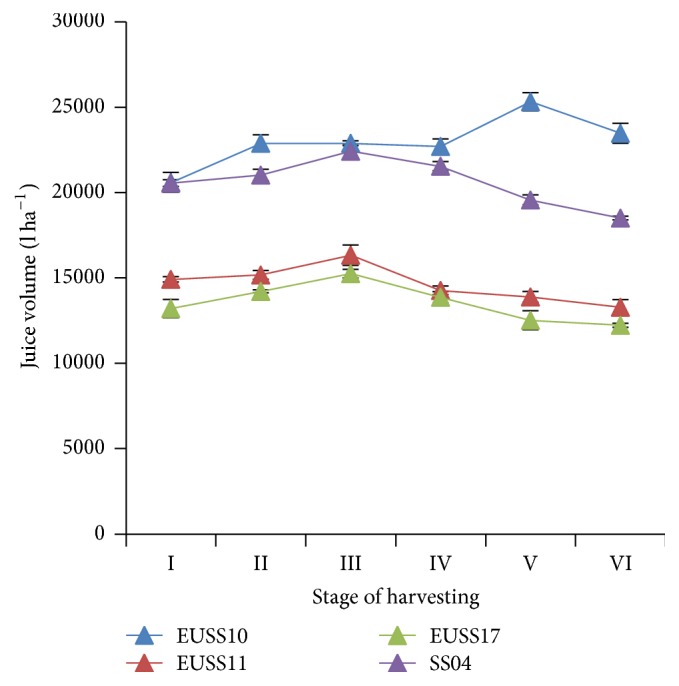
Effect of harvesting stage on juice yield of four sweet sorghum genotypes evaluated in part of western Kenya.

There was significant (*p* ≤ 0.05) difference in juice volume among all the genotypes (Table 3). Juice volume increased with maturity until stage III (21 days after flowering) and then declined thereafter (Figure 6). Juice volume also showed significant difference in the means for stages I, II, and III and also stages IV, V, and VI (Table 4). At the 21st and 28th day after flowering, most of the genotypes were at their peak in juice production. The highest juice volume was observed on line 35 days after flowering which is stage V.

**Figure 7:**
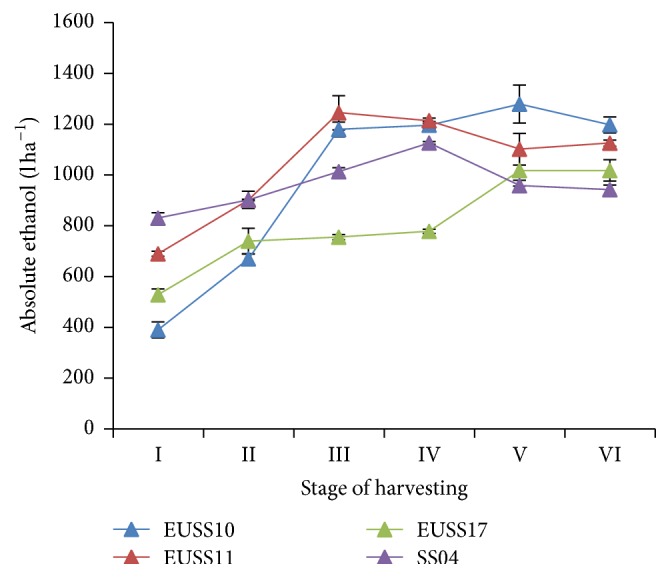
Effect of harvesting stage on ethanol yield of four sweet sorghum genotypes in western Kenya.

There was significant (*p* = 0.05) mean difference in the absolute ethanol volume for genotypes EUSS10, SS04, and EUSS11. However, there was no significant (*p* > 0.05) difference observed between EUSS11 and EUSS17 (Table 4). Mean difference in absolute ethanol volume for stages I (609.37 litres ha^−1^), II (803.07 litres ha^−1^), and III (1073.06 litres ha^−1^) was observed but there was no significant difference in stages IV (1078.63 litres ha^−1^), V (1089.08 litres ha^−1^), and V (1070.72 litres ha^−1^) (Table 4). Volume of ethanol obtained from all genotypes increased rapidly from stages I to III and then a decrease from stage III to stage VI. However, the decrease of ethanol observed in EUSS10 commenced after stage V. The volume was highest between stages III and IV except for EUSS10 which had the highest volume at stage V and then a drop (Figure 7). Among the sorghum genotypes, the mean volume of ethanol was (1062.78, 985.26, 961.96, and 805.96) litres ha^−1^ for EUSS10, EUSS11, EUSS17, and SS04, respectively (Table 4).

**Figure 8:**
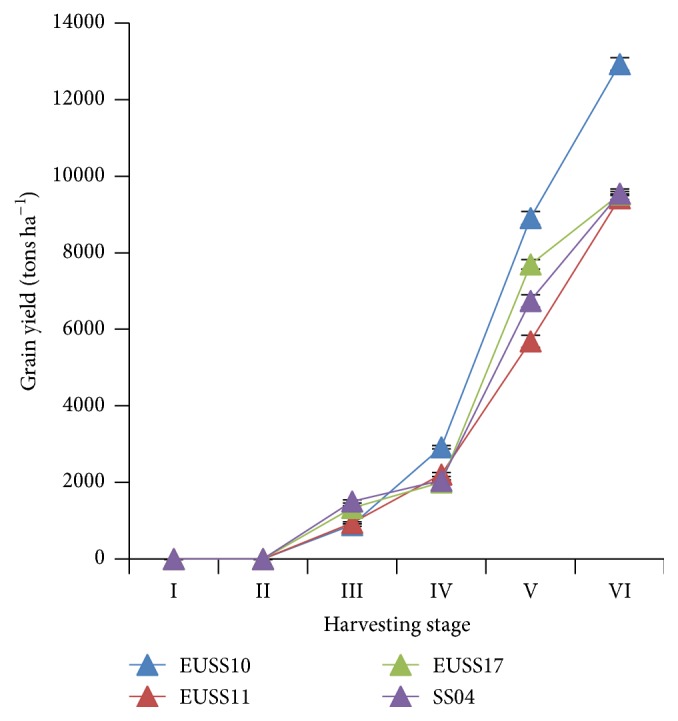
Effect of harvesting stage on grain yield among four sweet sorghum genotypes in western Kenya.

In this study, grain yield was a byproduct and not the main aim of sweet sorghum development. Significant interaction of genotype × stage indicates that ESS10 depicted higher means (1337.95, 2923.27, 8913.43, and 12936.79) tons ha^−1^ for stages III, IV, V, and VI, respectively, than the rest of the genotypes (Figure 8). There was significant difference in the grain yield among the genotypes and the stages of harvesting (Table 4). At stage I and stage II, there was no grain yield for all the genotypes. Significant grain yield was realized at the IV, V, and VI stage of harvesting (Figure 8). EUSS10 emerged as the most productive variety in terms of grain yield.

## 4. Discussion

The environment and soils in western Kenya highly favor the growth and development of sweet sorghum and the sorghum crops in general. Genotype SS04 is a sweet sorghum variety which was developed by ICRISAT. EUSS10 and EUSS11 have been released as sweet sorghum varieties while EUSS17 is still being developed by Egerton University. This study demonstrated that sorghum line EUSS17 reached anthesis stage the earliest but EUSS10 took the longest duration from sowing to flowering. The variation in the height, the girth, and flowering influenced juice volume content of brix and ethanol content from the four sorghum genotypes. Among the four genotypes tested, there were genotypic variations attributed to genetic background for most of the traits evaluated. Significant stage × genotype interaction suggests that stage of harvesting varies with sorghum genotype for girth, juice production, brix content, ethanol production, and grain weight. There were no differences in height probably due to environmental conditions that prevailed during growth period. Since accumulated biomass and cane yield is a function of height and girth, for improvement of sweet sorghum varieties, EUSS10 can be a good source of genes responsible for height as well as girth. EUSS10 had the highest volume among the genotypes at the 5th stage. The height at the flag leaf was significantly different for all the genotypes probably due to plant architectural and morphological differences.

EUSS10 produced the highest volume of juice at stage III of harvesting (Table 4) and in the stage III of harvesting (Table 4). This indicated that harvesting stage and genotype have an effect on juice volume. However, this study clearly demonstrated that volume of juice depends on the genotype, the size of the cane, and soil moisture related factors. The extracted juice contains the fermentable sugars that contribute to ethanol yield during fermentation process. High amount of juice volume together with content of brix directly impacts on ethanol production but should be balanced with accumulation of sugars which is predicted by the level of brix.

There was a rapid increase in absolute ethanol yield as the harvesting stage advances. This increase was directly related to increased brix and volume of the juice. Even though juice volume is an important aspect in bioethanol production, low brix can undermine the production of ethanol making brix an important quality trait in ethanol production. Brix has been seen to increase with increasing number of days from flowering time and also vary with the genotypes. Among the genotypes, EUSS10 had the lowest accumulation rate of the brix as well as the lowest* y*-intercept (Table 5). Often, there is variation in the sugar content among the genotypes at hard dough stage which corresponds to stage V [29]. In this study, it was evident that there was a decrease in the rate of brix accumulation in the stems of sweet sorghum varieties, and then it increased again in all the three genotypes. Although the content of juice in line ESS10 was generally high, concentration of brix which is important for fermentation was low. It is therefore important to determine inheritance and introgressed genes controlling high brix content into line EUSS10 from line EUSS11 and other genetic stocks. Any sorghum breeder with an objective of developing sweet stalk sorghum would aim at reconstituting genes for juice and large stem with those of accumulation of brix in the stem. This is because EUSS11 is high in sugar content while EUSS10 is high in juice volume.

The results from this study suggest that the rate of accumulation of sugar in the sweet sorghum stems decreases and then increases towards maturity. It is obvious that towards the maturity of sorghum plant, rate of accumulation of brix increases again. The physiological processes depend on the factors that support the productivity of the crop. Accumulation of sugar in the stems is influenced by several metabolic and transport processes as well as consumption within the sink cells [29]. The rapid increase in the rate of accumulation of the brix in sweet sorghum indicates that as the kernels mature, there is more carbohydrate retained in the stem of the plant, a factor that contributes to the concentration of the solutes in the stem hence increased brix. Also, there is reduction in the uptake of water and this was indicated by the reduction in the juice volume as from stages IV to VI.

Kernels obtained from sweet sorghum can improve the food situation among the rural households within the tropics. The results indicate that there was a significant increase in the weight of the kernels obtained per hectare for all the genotypes evaluated in this study. Determining the content of the juice and sugar in the juice and sorghum kernels was beyond the scope of this study. But a delay in harvesting by 14 to 21 days of the sweet sorghum would yield more kernels. Harvesting of sweet sorghum at stages III, IV, V, and VI would give ethanol yield which are not significantly different although for kernel production all the aforementioned stages are significantly different. From this it is hereby hypothesized that harvesting sweet sorghum at stage V would be appropriate for production of both ethanol and kernels. For the four sorghum varieties evaluated (EUSS11, SS04, EUSS10, and EUSS17), this would be between 92 and 117 days after sowing in western Kenya.

The chlorophyll content showed a steady decline with the stages of harvesting suggesting that it can be used to predict the time of harvesting of sweet sorghum. Even though each of the genotypes has its own level of chlorophyll at each stage, the same can be investigated to provide a way of detecting maturity of the sweet sorghum remotely using satellite images. Haboudane et al. [27] suggested that chlorophyll levels can be used to increase the precision in agricultural practices. It is imperative that harvesting stage of sweet sorghum can be predicted using chlorophyll levels. From this study, harvesting of sweet sorghum can be done when chlorophyll levels attains 20 to 40 for all the genotypes as measured by SPAD 502. This can be further investigated for accuracy and precision especially with the variation of locations.

## 5. Conclusion

Harvesting stage of sweet sorghum is best at the hard dough stage of the grain. Sweet sorghum is dual purpose crops that can be used for both energy and food security. Results from this study showed that harvesting sweet sorghum at stages IV and V (104 to 117 days after planting) would be appropriate for production of kernels and ethanol. However, these stages may be influenced by environmental conditions. The rate of sugar accumulation in the stems of sweet sorghum decreases and then increases towards maturity. From the study, the use of chlorophyll for prediction of the harvesting stage of sweet sorghum can be considered as a quick method of detection of sugar content in the stem.

## Figures and Tables

**Table 1:** Harvesting stage, duration after intrusion, and description of crop appearance.

Stage of harvesting	Duration (days)	Description of the crops
Stage I	7	Plants are at 50% flowering; panicles have no pollen
Stage II	14	Plants have all flowers and pollen shed
Stage III	21	Pollination is complete and grain filling begins: the milk stage
Stage IV	28	Grain filling is complete and grains begin to harden: soft dough stage
Stage V	35	Grains almost mature: the hard dough stage
Stage VI	42	The crops are at physiological maturity; the grains begin to dry

**Table 2:** Genotypes, equations, and first derivative of the functions for brix.

Genotype	Equation	*dy*/*dx* derivative of equation
EUSS10	*y* = 0.0134*x*^3^ + 0.157*x*^2^ − 0.665*x* + 2.558	0.0035*x*^2^ − 0.114*x* + 2.075
EUSS17	*y* = 0.1528*x*^3^ − 1.889*x*^2^ + 8.350*x* + 3.50	0.4584*x*^2^ − 4.696*x* + 12.588
EUSS11	*y* = 0.3634*x*^3^ − 3.999*x*^2^ + 14.709*x* − 1.333	1.0903*x*^2^ − 10.179*x* + 23.797
SS04	*y* = 0.2292*x*^3^ − 2.625*x*^2^ + 10.324*x* + 0.75	0.6876*x* − 6.609*x* + 16.166

**Table 3:** Mean squares of four sweet sorghum genotypes evaluated at different harvesting stages for agronomic traits: juice extraction, brix, ethanol, grain weight, and days to flowering.

Sources of variation	df	Overall height (cm)	Height at flag leaf (cm)	Cane yield (ton ha^−1^)	Girth (mm)	Volume of juice (l ha^−1^)	Brix	Absolute ethanol (l ha^−1^)	Chlorophyll	Grain weight (tons ha^−1^)	Days to 50% flowering
Genotype	3	805.01^*∗∗∗*^	4633.39^*∗∗∗*^	1344.94^*∗∗∗*^	47.33^*∗∗∗*^	492399033^*∗∗∗*^	235.29^*∗∗∗*^	18490974.51^*∗∗∗*^	342.51^*∗∗∗*^	6802524^*∗∗∗*^	2582^*∗∗∗*^
Stage	5	0.09	0.00	109.55^*∗∗∗*^	21.06^*∗∗∗*^	9213470^*∗∗∗*^	139.36^*∗∗∗*^	5755975.52^*∗∗∗*^	1933.89^*∗∗∗*^	296421650^*∗∗∗*^	0.67
Rep	3	887.97	1585.91	0.36	1.26	698318	1.79	124572.87	3.61	70922	0.58
Genotype*∗*stage	15	0.09	0.00	18.37	8.47^*∗∗∗*^	5350573^*∗∗∗*^	3.85^*∗∗∗*^	1337645.14^*∗∗∗*^	180.80^*∗∗∗*^	2724333^*∗∗∗*^	1.12
Error	69	76.13	129.38	10.00	0.67	580792	0.82	129130.10	7.29	26835	0.09
*R* ^2^		0.49	0.67	0.87	0.89	0.97	0.96	0.92	0.96	0.99	0.99

df: degrees of freedom.

^*∗∗∗*^Significantly different at *p* ≤ 0.001.

**Table 4:** Mean values of some agronomic traits and quality of juice and ethanol from sweet sorghum genotypes evaluated and harvested at different stages at Sugar Research Institute, Kibos, Kisumu, Kenya, in 2016.

Genotype	Overall height (cm)	Height at flag leaf (cm)	Cane yield (tons ha^−1^)	Girth (mm)	Volume of juice (l ha^−1^)	Brix (%)	Absolute ethanol (l ha^−1^)	Chlorophyll level	Grain weight (ton ha^−1^)	Days to 50% flowering
EUSS10	188^a^	167^a^	50.85^a^	20.79^a^	22976.9^a^	9.16^d^	1062.78^a^	46.54^a^	4277.06^a^	82^a^
EUSS11	182.25^b^	160.25^b^	45.39^b^	18.44^b^	20600.8^b^	16.21^a^	985.26^b^	44.13^b^	3428.52^b^	73^b^
EUSS17	179^bc^	149.75^c^	35.76^c^	18.11^bc^	14808.8^c^	15.29^b^	961.96^b^	40.42^c^	3308.06^c^	69^c^
SS04	174^c^	135.11^d^	35.73^c^	17.64^c^	13546.6^d^	13.91^c^	805.96^c^	38.06^d^	3046.30^d^	57^d^
Lsd	5.02	6.55	1.82	0.47	438.88	0.52	40.29	1.55	94.33	0.17

*Stage*										
I	181.02^a^	153.28^a^	43.45^a^	20.24^a^	17314.2^dc^	8.56^e^	609.37^c^	57.04^a^	0.00^e^	70.39^a^
II	180.83^a^	153.28^a^	44.84^a^	18.31^c^	18322.9^b^	12.56^d^	803.07^b^	50.97^b^	0.00^e^	70.39^a^
III	180.83^a^	153.28^a^	44.18^a^	19.28^b^	19220.8^a^	13.81^c^	1073.06^a^	45.14^c^	1170.10^d^	70.39^a^
IV	180.83^a^	153.28^a^	41.17^b^	16.80^d^	18100.4^b^	14.18^c^	1078.63^a^	40.43^d^	2295.99^c^	70.39^a^
V	180.83^a^	153.28^a^	39.40^bc^	18.79^b^	17821.0^bc^	15.56^b^	1089.08^a^	30.75^e^	7262.43^b^	70.39^a^
VI	180.83^a^	153.28^a^	38.55^c^	18.86^bc^	17120.4^d^	17.18^a^	1070.72^a^	29.39^e^	10362.89^a^	70.39^a^
Lsd	6.15	8.02	2.23	0.57	537.52	0.63	49.35	1.90	115.54	0.21

Means designated by the same letter within columns are not significantly different at *p* = 0.05.

**Table 5:** Estimated linear regression parameters of four genotypes of sorghum on the rate of response of chlorophyll content and brix to six harvesting stages.

Genotype	Equation	Slope	*y*-intercept	*R* ^2^
EUSS10				
Chlorophyll	*y* = −2.217*x* + 51.781	−2.217	51.781	0.872
Brix	*y* = 0.986*x* + 5.633	0.986	5.6333	0.857
EUSS17				
Chlorophyll	*y* = −5.082*x* + 54.518	−5.823	54.518	0.908
Brix	*y* = 1.507*x* + 10.017	1.507	10.017	0.890
EUSS11				
Chlorophyll	*y* = −8.287*x* + 75.537	−8.287	75.537	0.975
Brix	*y* = 1.907*x* + 9.533	1.907	9.533	0.850
SS04				
Chlorophyll	*y* = −8.937*x* + 71.691	−8.937	71.691	0.942
Brix	*y* = 1.529*x* + 8.567	1.529	8.567	0.868
